# List of medicinal plants from Epigraphia Indica

**DOI:** 10.6026/97320630016695

**Published:** 2020-09-30

**Authors:** J Sridevi, J Sriram, T Saket Ram, PVV Prasad, K Kanakavalli

**Affiliations:** 1 National Institute of Indian Medical Heritage, Hyderabad - 500036, India; 2 Central Council for Indian Medicine, New Delhi, India; 3 Central Council for Research In Siddha, Chennai, India

**Keywords:** Plants, Epigraphia Indica, Inscriptions, Archeological survey of India

## Abstract

It is of interest to list plants and its usage in olden days. The history of plants is closely related with the history of medicine since time immemorial. Various literature data shows the usage of medicinal plants. It is linked to various system of medicine like Siddha, Ayurveda and Unani. This is in practice for more than 5000 years. People in olden age had a detailed knowledge on these plants. Hence, we collected such data from the first five volumes of Epigraphia Indica, which was published, by the Archeological survey of India from 1882 to 1977. These data provides a framework on various plants and its usage in various ways, in different time period in human history. This helps us to drive valuable bio-medical information for application in modern scientific biomedical methods. We further plan to sort and glean such data so as to help in evaluating the total biomedical data in Epigraphia Indica to extract useful biological knowledge for application in evidence-based biomedicine.

## Background:

An epigraph on the stone named as inscriptions commonly gives us historical record, which is maintained for the future generations. Epigraphy is the science of studying, analysing and interpreting inscriptions found on stones, rocks, artefacts, idols, walls and other structures of ancient times. Epigraphia Indica was published by the Archeological survey of India from 1882 to 1977. It comprises of about 43 volumes. James Burges edited the first two volumes in the year 1882 and 1894. He was the founder of Indian Antiquary in 1872. The Vol III (1894-95), Vol. IV (1896-97) and Vol. V (1898-99) was edited by E. Hultzch a German Indologist and Epigraphist who was known for his work in deciphering the inscriptions of Ashoka [https://en.wikipedia.org/wiki/ E._Hultzsch]. Epigraphia Indica gives ample information on geography, religious systems, affliations of families and dynasties, taxes, land tenures, magistrates, customs, manners, organization of societies, language, and system of writing of ancient times.

The 5 volumes of Epigraphia Indica were collected from the Medico-historical library of National Institute of Indian Medical heritage, Hyderabad [1-5]. It is unique with the collection of more than 10,000 books, which include very rare publications that date back to the 15th century on History of Medicine & allied subjects in various languages, which serves as a referral centre for research in medicine.

## Epigraphia Indica - Volume 1 (1892) Reprinted 1983 (Table 1 - see PDF) Mandara, santana and kalpa trees (Badaun Stone Inscriptions of Lakhanapala, Page no: 62)

The inscription, which holds the name of mandara, Santana and kalpa trees, is currently present in the Lucknow Museum. It was found in August 1887, in the ruins of the south gate of the old fort of Badaun, the chief town of the Badaun district of the North - Western provinces. According to Prof. Kielhorn the characters are devanagari with a letter size 5/8'' to 3/4'' and it belongs to the 12th or the 13th century A.D. It is interpreted that the princes born in the Rastrakuta family protected the town in a great manner which flourished with prosperity and radiant having wealth. The land was surrounded by the excellent flowers of mandara trees resembling the city of Indra's paradise and also Kalpa, Santana trees pleasing with its lofty purity of the deities as the seat for the assembly of the immortals.

## Salt, oil, betel, pepper, ginger, vegetable (Bilhari Chedi Inscription, Page no: 270)

The stone, which bears this inscription, is said to have been found at Bilhari, described as the oldest towns in the Jabalpur district of the central provinces. In 1861, it was at Jabalpur, where it was reported to have been carried out about 20 years before and it is now in the Nagpur museum. The characters are Nagari of about the 11th century. The average size of letters is 3/4'. The salt, oil, betel, pepper, ginger, vegetable along with some money was given to traders for every Elephants and horses they bought.

## Information on various plants (Cintra Prasasti of The Reign of Sarangadeva, Page no: 277)

The inscriptions in the black stone containing 66 lines according to the Prof. Bhuler belonged to a temple at Somanatha or Devapattana in Sorath. The inscription was composed in honour of the consecration of five Lingas particularly Tripuranthaka, erected at the place Sorath. Dhavala the period of Chalukya kings of Gujarat, who were descended from the Vyaghrapalliya or Vaghela branch, founded it. The alphabet is the common Nagari of the thirteen-century. According to verse 47, in order to cleanse the Gods daily - two kavadis of water and a broom of Zizyphus jujube for sweeping the buildings. The things are particularly procured by the clever pupil named as batuka in return for the use of the naivedya food and the money for his monthly expenses. Every month eight drammas for sandalwood in the madraka was purchased for tripuranthaka the God who bears the cresent of the moon on his head and the gardeners furnished two hundred white roses and two thousand fragrant oleander blossoms. Two manakas of husked rice (chosa) and one manaka of Phaseolus mungo, four karshas of clarified butter, and as much oil for the lamps. That constant man provided a judge of the merits of others, five betel nuts of good quality, daily in the store- house. For offering incense every month two manas of fragrant gugglu was provided. Pan-supari called as bitaka is the little three-cornered parcel of ground arecanut, lime etc., which is wrapped up in a betel pepper leaf and hold together by a clove stick into the leaf. One manaka of rice and two pallikas of Phaseolus mungo, as well as two karshas of clarified butter, must be daily given to the pasupala for the offerings.

## Epigraphia Indica -Volume 2 (1894) (Table 2 - see PDF) Punnaga and betel nut (Badal Pillar Inscription of the Time of Narayanapalla, Page no. 194)

A slab of stone with inscription was found in Nagpur museum contains 40 lines which covers a space of 4' 51/2'' broad by 2'81/4'' high. The characters are Nagari, which belongs to beginning of 12th century A.D according to Prof.Kielhorn. It accounts to the Paramara rulers of Malava. Here Punnaga and Betel creepers were referred to show the multitudes of distinguished warriors, which also add to show his fame.

## Epigraphia Indica volume 3 (1895) (Table 3 - see PDF)Areca nuts, jack-fruits, plantains, cocoa-nuts and mangoes:(Ranganatha Inscriptions of Sundara-pandya, Page no. 15)

The following plants name was found in the inscription, which was found on the east wall of second prakara of the temples of Ranganatha on the place of Srirangam inTrichinopoly taluka. It belongs to the time of Sundara-Pandya who ascended the throne in 1250 or 1251 A.D according to Prof.Keilhorn. It was in Sanskrit and Grantha script. Different kinds of fruits, which was manufactured out of heaps of gold, such as Areca nuts, jack-fruits, plantains, cocoa-nuts and mangoes delighted lord Murari (Vishnu). Dhimara (Adhimara) trees, karaka trees(Achyutapuram Plates of Indravarman, Page No. 130) The copper plate with the reference of dhimara and karaka trees was found in the city of Kalinganagara. The land has the boundaries with dhimara trees and karaka trees. Nobody shall cause hindrance if he opens the sluice of the tank.

## Tinduka tree, kadamba tree, jamboo tree (Chicacole plates of Devendravarman - the year 183, Page No. 134)

According to Prof. Hultzsch the word has its reference in the copper plate, which was found at Chicacole of the Ganjam district. It was in Nagari forms. Along the boundaries of the village these trees are planted. Jack fruit (Spurious sudi copper-plate grant purporting to have been issued by butuga in Saka-Samvat 860, Page no. 184) The characters with the old kanarese alphabets with the average size of 3/16 purports to be a record of that western Ganga prince Batuga who according to the atakur inscription slew the Chola king Rajaditya, in the war between the latter and Rashtrakuta king Krishna III, in or just before 949-50 A.D. On the boundaries of the land jackfruit tree is planted.

## Tanri-endal(Parla-kimedi plates of the time of Vajrahasta, Page no. 226)

The inscription has the reference, which is found in the chief town of the Parla-Kimedi Zamindari in the Ganjam district of the Madras Presidecy. The characters were in the Nagari with the average size of about 1/4''. According to Dr. Burnell the alphabet belongs to the year of 608 A.D. This is found in a hill on which tanri trees grow.

## Epigraphia Indica - volume 4 (1896-97) (Table 4 - see PDF)Madhuka tree (Nadagam plates of Vajrahasta Saka – samvat 979, Page no. 188)

One Sanku Appana found the reference holdimg the inscription in Narasannapeta taluka of the Ganjam district,, a cultivator, while he was working in the field. It is believed by the villagers that the plates belonged to some Jangams, a sect of Saivas, who had been living in this locality until 50 years ago according to author G.V.Ramamurti. Madhukesa the name given after the madhuka tree.

## Mandhara tree (Pitahapuram inscription of Mallapadeva Saka-Samvat 1124, Page no. 240)

The inscription holding the reference is found at the entrance of the Kunti madhava temple at Pithampuram. The languages of the inscriptions are Sanskrit and Telugu prose. Youger brother,Vimaladitya an ocean of honour, the mandara tree on earth who was not treacherous even towards an enemy whose weapon was his arm ruled the earth for seven years.

## Kalpa tree (Pitahapuram inscription of Mallapadeva Saka-Samvat 1124, Page no. 240)

The inscription holding the reference is found at the entrance of the Kunti madhava temple at Pithampuram. The languages of the inscriptions are Sanskrit and Telugu prose. Rajara the ornament of the race of the moon the kalpa tree on earth ruled Andra-mandala for forty years. Tamarind tree, palmyra tree, banyan tree (Nandamapundi grant of Rajaraja I dated in his thirty second year 1053 A.D, Page no. 303). The copper plate inscription, which holds the reference, is currently present in the madras museum. The average size of the letters is about 1/4'' and the language is Sanskrit and telugu. On the borders of the village tamrind tree, palmyra tree, banyan tree was planted.

## Tamarind tree, banyan tree, olive tree, papal tree (Kadaba plates of Prabhutavarsha; Saka-samvat 735 A.D, Page no. 349)

The above reference is in the copper plate inscription, which is found at Kadaba in the Tumkur district of the Mysore state and currently in Mysore Government museum, Bangalore. The language is both in Sanskrit and Kanarese. Along the bounadaries of the village it was planted.

## Epigraphia Indica – volume 5 (1898-99) (Table 5 - see PDF) Saka trees (The Asoka edicts of Paderia and Nigliva, Page no. 2)

The stone inscription was from Nepal Terai, where Nigliva is situated 38 miles northwest of the Bengal. According to the author the language is the Magadhi of the third century B.C. There was a great grove of saka trees (Tectona grandis) on the bank of the lake situated on the slopes of the Himalaya. Jack fruit (Yekkeri Rock inscription of the time of pulikesi II, Page no.9) The inscription was found in the Yekkeri a village in Belgaum district. The language is in Sanskrit. The inscription refers itself to the reign of the Western Chalukya King Pulikesi II. In the town Agariyapura, jackfruit is found.

## Betel-leaves (Inscription at Managoli, Page no. 23)

Managoli is a village of Bagewadi taluka of the Bijapur district. The writing of this record covers an area about 2'10'' broad by 4'6 1/2' high and beongs to 1161 A.D. The characters are Kanarese. The Gutrigas gave fifty betel leaves per honnu on the betel leaves that they sold. Various plants name (Pitahapuram plates of Virachola, Page no. 100) The copper plate of inscription from Pithampuram has the reference both in the telugu and Sanskrit language. The only copperplate inscription of Vira-Chola which has been published before are the chellur plate of his 21 st year. Babul tree, nimba tree, wood apple tree, bhillu tree, tamarind tree, palmyra tree, kshira tree, svarnapushpi tree (Dibbida plates of Arjuna, Page no. 109)

The copper plate inscription is found in the village of Dibbida Agraharam, in the Viravilli taluka of the Vizagapatam district. The characters are intermediate between those of the latest Eastern Chalukya inscriptions and Telugu characters. The size of the letters varies between about 7/16'' and 3/16''. The language is in Sanskrit. The inscription records the date of Saka Samvat 1191. These plants were planted along the boundaries of the village.

## Water lily (Inscriptions at Ablur, Page no. 237)

The inscription is a record of the time of the Western Chalukya king Taila III. The size of the letter ranges from 3/8'' to 3/4''. The language is in Kanarese. The plant, which belongs to kadamba family, is offered to God.

## Conclusion:

The use of Indian MEDICINAL PLANTS is well documented in Siddha, Ayurveda and Unani. Therefore, it is of interest to collect data from the first five volumes of Epigraphia Indica published by the Archeological survey of India from 1882 to 1977. Historical records confirm the importance of plants in previous centuries throughout different ruling periods. The usage of those plants is exhibited in different manner lie the offerings to deities and plantation of such tress along the boundaries, etc. We further plan to sort and glean such data so as to help in evaluating the total biomedical data in Epigraphia Indica to extract useful biological knowledge for application in evidence-based biomedicine.
